# Supplementary figures and images for: Kinome inhibition states and multiomics data enable prediction of cell viability in diverse cancer types
Source: PLoS Comput Biol. 2023 Feb 21;19(2):e1010888. doi: 10.1371/journal.pcbi.1010888 (PMC9983880; doi:10.1371/journal.pcbi.1010888)

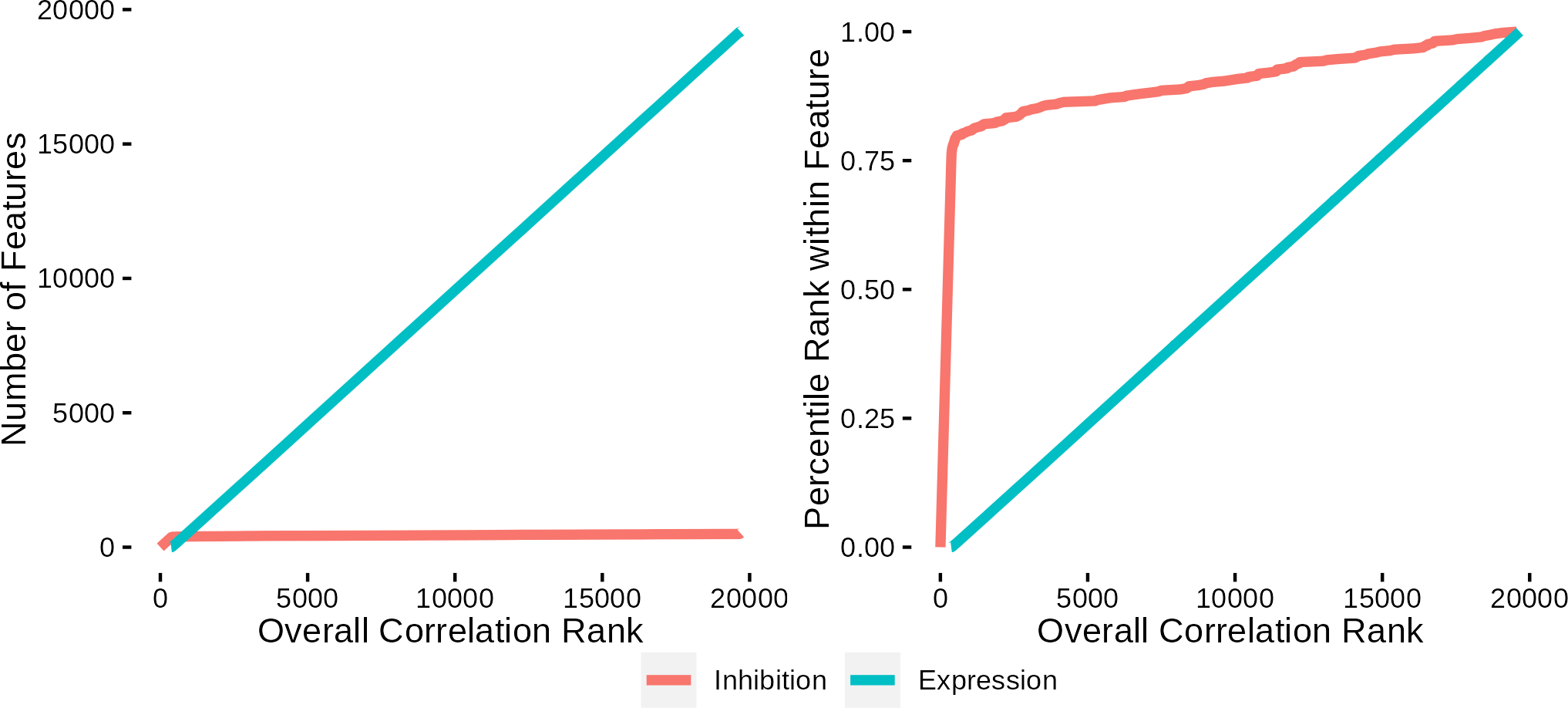

Supplement: S1 Fig — Extended version of Fig 2D covering all correlation ranks. (TIFF) [file pcbi.1010888.s001.tiff]

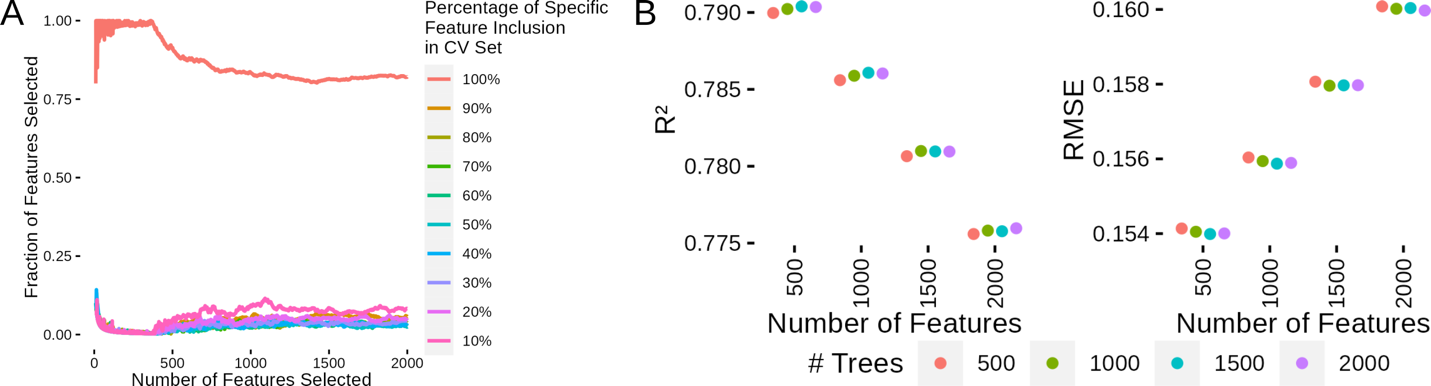

Supplement: S2 Fig — (A) The effect of cross validation data division on which features are selected for model inclusion. (B) The effect on R2 and RMSE of increasing the number of trees used in the random forest algorithm. (C) The effect on R2 and RMSE of modifying the selected predictor count and the minimal node size used in the random forest algorithm. (D) The distribution of R2 and RMSE for single compound or cell line cross validation results. (TIFF) [file pcbi.1010888.s002.tiff]

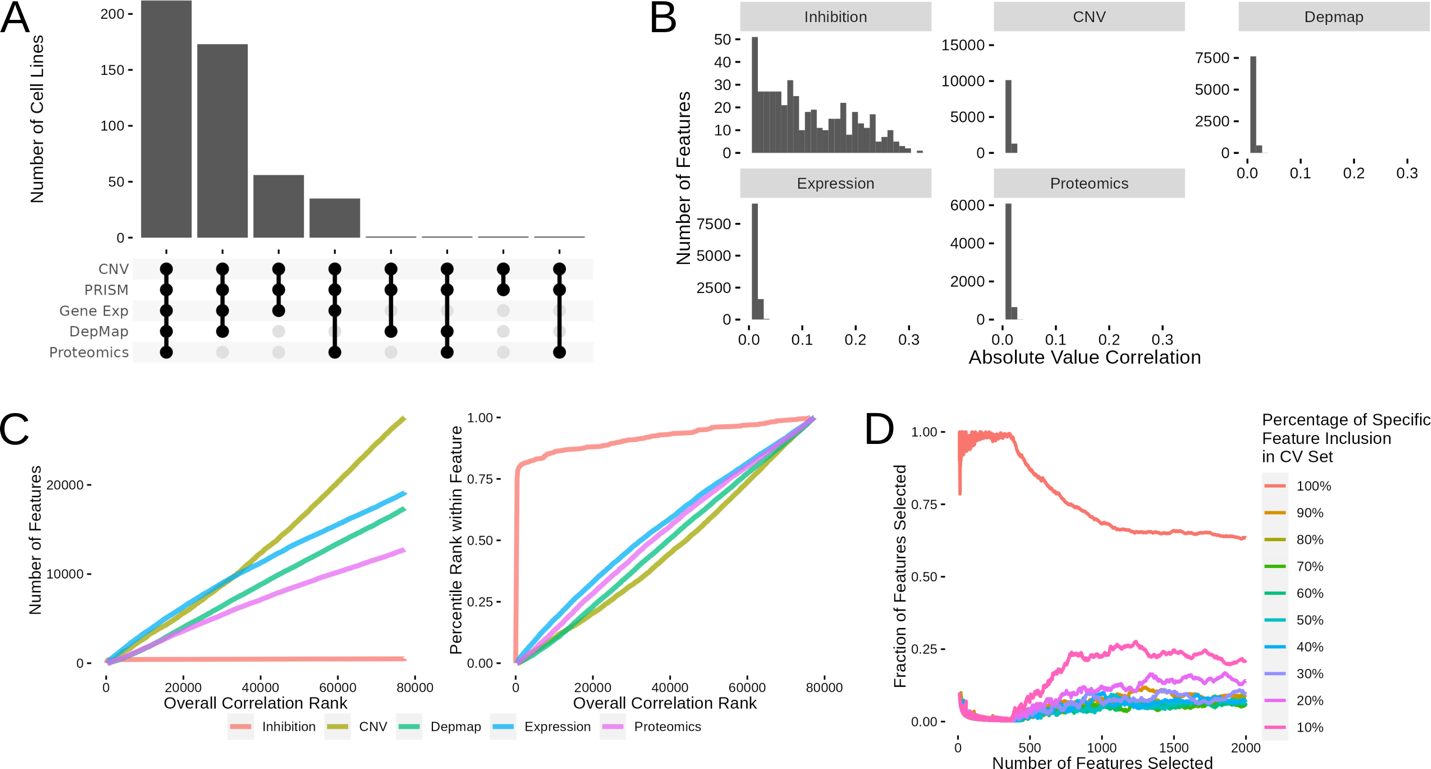

Supplement: S3 Fig — (A) Upset plot showing the overlap between data sets across cell lines in the PRISM assay. (B) Small multiples plot showing the correlation of individual features to imputed cell viability for each of the feature types considered in this model. (C) Full feature correlation rankings for all data set types considered for Fig 5. (D) Effect of random 10-fold cross validation subsetting on which features are included in what percentage of the cross validation data sets. (TIFF) [file pcbi.1010888.s003.tiff]

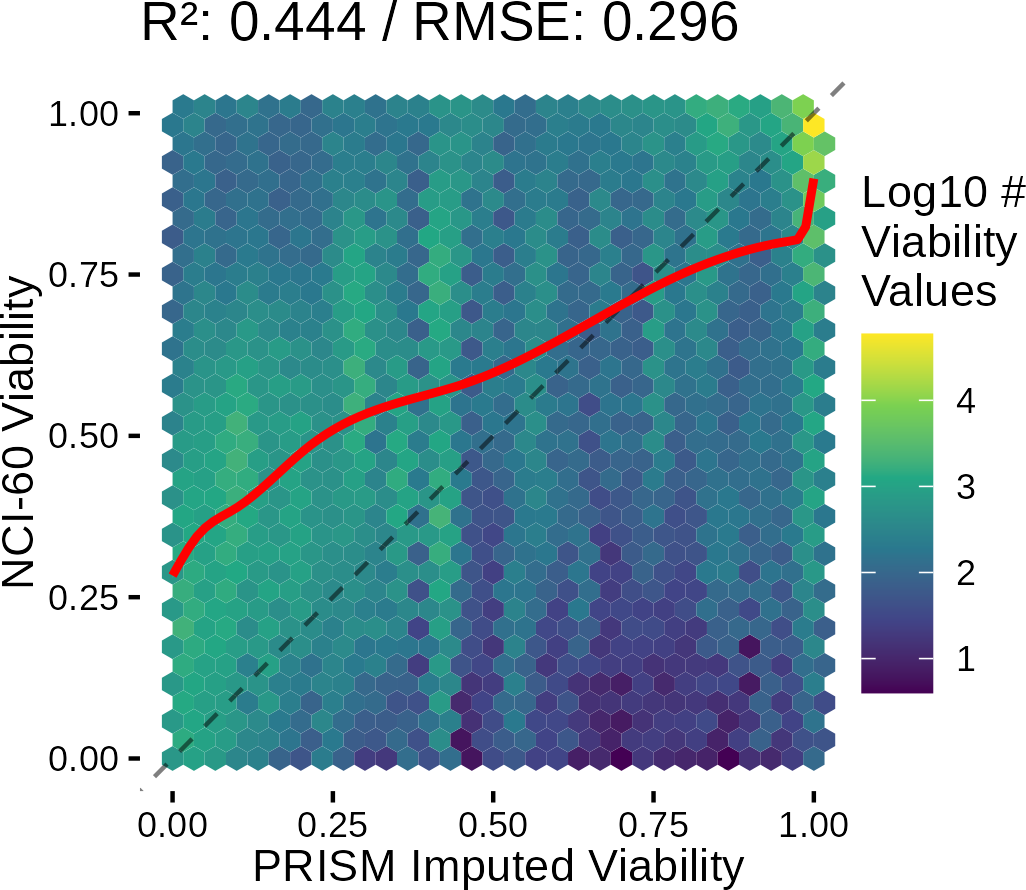

Supplement: S4 Fig — The red line shows a Loess fit through the data set. (TIFF) [file pcbi.1010888.s004.tiff]
