# Supplementary material for: Kinome inhibition states and multiomics data enable prediction of cell viability in diverse cancer types
Source: PLoS Comput Biol. 2023 Feb 21;19(2):e1010888. doi: 10.1371/journal.pcbi.1010888 (PMC9983880; doi:10.1371/journal.pcbi.1010888)
Supplement: Attachment — Submitted filename: ResponseToReviewers.pdf [file pcbi.1010888.s006.pdf]

Joint Department of
BIOMEDICAL
ENGINEERING
UNC CHAPEL HILL
NC STATE UNIVERSITY

10010 Mary Ellen Jones Building
Campus Box 7575
Chapel Hill, NC 27599
919.966.1175 • bme.unc.edu

4130 Engineering Building III
Campus Box 7115
Raleigh, NC 27695
919.515.5252 • bme.ncsu.edu

August 12, 2022

Response to Reviewers for “Kinome Inhibition States and Multiomics Data Enable Prediction of Cell Viability in Diverse Cancer Types”

We would first like to thank the reviewers for their thorough examination of our paper. Both reviewers' thoughtful comments have helped clarify and improve the paper. Our responses to points brought up follows below.

Reviewer #1: The recent development of cancer treatment emphasizes targeted therapies. Protein kinases represent one of the largest classes of anti-cancer targeted therapies. Therefore, uncovering the correlation between kinome activity and cancer cell viability via regression model could provide a list of potent drugs based on predicted cell viability value ahead of treatment and infer potential key kinases in regulating cell viability. Berginsiki et al set out to determine whether a combination of kinase inhibition profiles at multiple doses and gene expression could predict the effect of kinase inhibitors on cancer cell viability. They integrated kinase inhibition profile data and gene expression data of different cancer cell lines to build a random forest model to predict cell viability. The final model achieved R2 value as 0.79 in 10-fold cross-validation. This manuscript compared linear and non-linear models, utilized feature ranking via Pearson’s correlation, and introduced data from various aspects like copy number variation and proteomics as additional input for model training. Besides developing the model, this manuscript showed some insights from the model itself, pointing out that most top-ranking features are from kinase inhibition data and that genes selected as model features have a high possibility of interacting with kinase. This manuscipt also considered the experimental variation and evaluated the difference between the original PRISM value and lab assay result before validating the model results on new compound–cell line combinations.
Major comments
1. The conceptual bias of training data. The kinase inhibition profiles comes from Klaeger et al, whose data is derived from a mixture of four cell lines, while cell viability and gene expression data (PRISM) are from a panel of over 400 cancer cell lines. The combination of kinase inhibition state from a mixture of cell lines and cell line-specific gene expression data might not be a proper input to train a model predicting individual cell line viability. Have the authors tried to use kinase inhibition profiles from gold standard radioactive assays which could avoid in vivo bias due to cell line variation? For example, GSK kinase inhibitor profiling or HMS LINCS profiling done at multiple doses.

We also had similar questions about the applicability of the Klaeger cell line mixture data to a data set as broad as PRISM when we initially started this project. Only once we saw how the broad coverage afforded by the Klaeger et al data allowed us to build predictive models were we convinced by the general usefulness of this data. Our primary justification for why we think the models perform as well as they do is that the kinobead mass-spec based assay used by Klaeger produces kinase inhibition profiles that are applicable to a wide range of kinomes present in each of the cancer cell lines in the PRISM collection. To help clarify this, we’ve added this sentence to the introduction:

> This work was conducted using a lysate mixture derived from four cell lines which provided a broad representation of the kinome.

And also added this phrase to the discussion:

> derived a proteomic assay based on a a four cell lysate mixture

We believe the second point concerns whether alternative methods for assessing kinase inhibitory states, such as through KinomeScan or radioactive assays, could also be used to successfully predict cell viability. We definitely believe so, though each approach and associated data type has it's own specific properties. For KinomeScan assay data sets, we feel that this type of data is different enough from the Klaeger et al collection, that we are currently building a second paper around assessing KinomeScan data. Differences can be significant, for example for this data, we can perform IC50 predictions but not whole dose-response curves as is done in this work. That paper is still being written, but our preliminary results indicate that cell viability models can also be successfully built using this data as well. We know of at least one older study (Anastassiadis T, et al. 2011. Nature biotechnology 29:1039–1045) using recombinant kinases and radio-labeled ATP to try and characterize kinase profiles. Like KinomeScan data, it has its own properties that have to be assessed. Longer-term, we do hope to combine and compare these different data types to better assess their strengths and weaknesses when used in similar modeling approaches.

2. Lack of innovation from a modeling perspective. To build the model, Berginsiki et al applied linear regression, random forest, and XGBoost with a limited parameter tuning process. Introducing a neural network with different transformers might improve the performance for large-scale multi-dimensional data. To optimize the random forest model, there should be parameters other than tree number to adjust, like subsample size, max leave, depth and child weight, etc. Show optimization of RF. Is it possible to make some more improvements to this model if including these features in parameter tuning, such as using grid search or randomized search for 1000 iterations or so?

While we find it extremely interesting and have work underway exploring this, we were not intending to focus this effort on creating a new modeling approach for predicting cell viability or other cellular phenotypes. We believe that the majority of the innovation described in this paper comes from the identification of a novel data type - proteomic kinase inhibitor profiles - as a powerful, highly-informative molecular measure, with significant potential in downstream modeling efforts. While not having generally been leveraged, the use of inhibitor profiles appears to have great value, as drugs largely target and exert their effect at the protein level, so one would expect that measurements at the protein level would potentially have something "unique" to say about cellular responses to a drug that are not captured through transcriptional, mutation or other genomic measurements. This is what was observed in this work, where inhibitor profiles were by far the single most informative data type.
We also recognize that newer neural network based models may eventually prove to have the best performance on these problems, and we have now also tested Tabnet (https://arxiv.org/abs/1908.07442) on our data set. Tabnet is an attention-based neural network which focuses on tabular data and performs roughly equally to XGBoost in this context (see updated Figure 3A). We hope that this additional model type provides an additional data point concerning the performance of just one type neural network-based model. You are correct that there are other parameters that can be adjusted on the random forest. We started with tree count as we thought it would have the largest impact and did not see any dramatic improvements. We then focused more on exploring the effect of other data types. You are also correct that some of the other parameters might have effects on model performance, so we have gone back and also tested the effect of modifying the minimal leaf node size and the number of predictors selected at each branch. Neither of these parameters had a dramatic effect on either model metric (new supplemental figure 2). Testing these twelve new parameter sets took about 46 hours of wall clock time on a 16-core machine (primarily due to having ~500,000 data points to sort through), so we won't be able to expand this hyperparameter search any further in this paper.
As also mentioned in our response to a question from Reviewer 2, we could have considered many more models/algorithms along with associated parameter tuning. We expect that there are likely several algorithms

that will be able to provide similar performance to the random forest. We are actively looking into novel method development, but in this work we were not trying to find the “best” model and instead focused our attention on the contribution of different data types.

3. I applaud the author's effort to examine the difference between original PRISM data and lab assay results, and there exists a similar trend between model prediction curves and lab assay results, however, R2 score of 0.5 is merely reasonable. Moreover, this comparison is based on the assay results and the model prediction values, which could not infer how the model might perform on the original PRISM data, given the observed difference between PRISM value and assay result.

We thank the reviewer for their kind words about our assessment of the well-known reproducibility constraints that exist around cancer cell line screening data(Mullard 2021; Errington et al. 2021). As an initial clarification for:

“Moreover, this comparison is based on the assay results and the model prediction values, which could not infer how the model might perform on the original PRISM data”

We would like to point out that our metrics for how the model performs on the original data are included in figures 3A and 3B, where we employ a 10-fold randomized cross validation scheme on the original data. We agree that our final R-squared score of ~0.5 is definitely lower relative to the nearly 0.8 achieved in our other comparisons. Recognizing this, we sought to understand if this was due to model performance issues, or whether there were assay artifacts or technicalities that prevented us from effectively reproducing a high-throughput cell viability assay.
First, our results in figure 6C indicate that for exact compound-cell line combinations in the PRISM dataset, we have a “ceiling” of ~0.5 R-squared in simply reproducing cell viability measured by the PRISM assay. Second, to further investigate this difference, we sought to investigate the variance associated between replication of the same experiment by different experimental groups. To do this, we turned to a popular large screening dataset of 60 cancer cell lines, provided by the NCI-60 resource. We then compared the cell viabilities measured by PRISM with the NCI-60 dataset, matching the datasets by compound, cell line and nearest dose measured. The PRISM cell viability vs NCI-60 cell viability comparison shows an R-squared value of 0.44, across 172 compounds, 32 cell lines, and all doses measured. Thus, replication of a dose response curve by different groups is showing as much (or even greater) variation than our predictions are with experimental results.
Given that this degree of experimental agreement between two specialized, high throughput screening databases is similar to the agreement between our lab assay results and the model predicted results (~0.5 R-squared), we feel that the moderate 0.5 R-squared result we obtained is quite reasonable and “within the range” of variation that is being observed by any method - experimental or computational. We have attached this comparison between PRISM and NCI-60 as supplementary figure 4.

Reviewer #2: Summary:

In this study, the authors develop a set of predictive models for cell viability across varying concentrations of kinome inhibitors, using kinome "inhibition states" as predictive features. The work builds on a 2017 paper by Klaeger et al. which profiled 243 kinase inhibitors to identify potential off-target/polypharmacology effects. This study integrates the kinome profiles from Klaeger et al. with -omics data from CCLE and PRISM, showing that kinome profiles tend to be more informative in viability prediction than baseline gene expression values, and additionally that kinome profiles and gene expression can be combined to produce slightly better models than either data type alone. The authors validate their modeling strategy on held-out kinase inhibitors and cell lines, showing that for a subset of breast cancer cell lines their models tend to generalize well.
The paper is clear and well-written, and the experiments provide compelling evidence that kinome inhibition profiles contain predictive signal for cell viability screening of kinase inhibitors. The validation on breast cancer cell lines and the predictions across all of PRISM will serve as a useful resource, and the GitHub repository containing the code is well-documented and easy to navigate. However, there are some aspects

that could be explored further to ensure the robustness of the study's main conclusions, particularly with relevance to existing work in viability prediction.
Major comments:

1. The conclusions of the authors in this study seem slightly at odds with previous work, particularly Dempster et al. 2020 (https://doi.org/10.1101/2020.02.21.959627) which found that baseline expression was generally effective for predicting cell viability for various types of perturbations, moreso than genomic profiles. Importantly, the studies are looking at different datasets and drug classes: the Dempster et al. study looked at a broader set of compounds rather than just kinase inhibitors, and at GDSC and several other datasets in addition to PRISM. However, the application to cell viability and overall conclusions of both studies still seem quite related.

Given the seemingly contradictory conclusions of these two studies, I think the question I would be most interested in is whether this is a biological difference (i.e. kinase inhibitors behave differently than the larger set of compounds as a whole), or whether a difference in experimental design is causing the discrepancy (e.g. model setup, label definition, cross-validation splitting strategy, etc, all of which have subtle differences between the two studies). Glancing through the methods of both papers I noticed a few differences - perhaps most notably the Dempster et al. study uses only the PRISM dose with the most variance over cell lines for each compound, while the present study looks at multiple doses as described in the Methods section and in Klaeger et al.

It would be informative if the authors tried running their gene expression pipeline on their set of kinase inhibitors with the labeling strategy (selecting a single representative dose) described in Dempster et al. - if the results are unchanged, it would inspire more confidence that the set of kinase inhibitors studied in this paper are indeed biologically different from the rest of the compounds tested in the Dempster et al. study, rather than reflecting an experimental design difference. If the results are different, this would also be a noteworthy outcome - it would support the idea that looking at multiple doses, rather than picking a single representative dose, is a necessary/important difference between the studies.

This is an excellent point and thank you to the reviewer for bringing this paper to our attention. You are correct that the largest difference between the studies is that Dempster uses only a single dose for model construction, though this work also generates a single model for each compound (as opposed to our single model). To make a comparison possible, we've taken your advice (following from Dempster) and subset the PRISM compounds we worked with to the single highest cell viability variance dose by compound. We then re-ran the feature selection and random forest modeling on these new data sets. Overall, it appears that the kinase inhibition states are still the most informative within the model. There are two new subpanels in Figure 4 showing these results and an additional paragraph in the "The Combination of Kinase Inhibition States and Baseline Gene Expression Produces the Best Predictions" that describes this in more detail. The single dose models were not as accurate as the models produced with the full range of concentrations. This isn't completely surprising as knowing only the compound dose already produces a R^2 of 0.49, so it appears that the additional information provided by each dose is critical for the full model performance.
We do not think the papers are really at odds, however, as we do agree that RNA expression is highly valuable within our model. We think of the kinase inhibitor profiles as providing a cell-line agnostic initial "state" that is expected to be induced by inhibitor treatment, with the RNA expression altering that state in a cell-line-specific manner (and thus the improvement in predictions with its inclusion). Direct comparisons are also challenging as the two papers are trying to predict different targets (single vs all dose prediction, comparison relative to DNA-based features in Dempster, etc.). We thus think of this work as being complementary to the findings of Dempster et al, and we have cited this in the manuscript.

As for the overall differences between kinase inhibitors and the rest of the compounds in Dempster, our working theory is that if this type of rich proteomic targeting data was available for all compounds, it would similarly be valuable for other classes of compounds. We hope this addresses your point about biological differences in the context of compound perturbations, but this is a fascinating set of questions none the less. Thanks once again for pointing us towards this paper, we think the additional single dose modeling has further enhanced the paper.

2. Related to the first point, the example of BRAF inhibitors in Figure 6 of the Dempster et al. paper (dabrafenib and vemurafenib) could be a useful positive control, or example to use to examine the differences between the studies. Their data suggest that baseline RXRG expression, particularly in melanoma cell lines, is a strong univariate predictor of response to dabrafenib and vemurafenib - is that still the case for the authors' pipeline (i.e. are BRAF inhibitors a class of drugs where gene expression is particularly effective, and the poor performance shown in Figure 4 is mostly driven by other cell lines or kinase inhibitors), or are the results different when the labels for multiple doses are introduced? Does the authors' multivariate gene expression-based model perform well for BRAF inhibitors?

This is an interesting finding that our models are not designed to detect. Baseline expression of RXRG doesn't appear in the final model. Our guess is that RXRG has a strong impact on melanoma combined with dabrafenib and vemurafenib, but this specific effect is washed out in the PRISM subset we're working with. We are looking at extending this work to focus on each of the ~27 cancer types that are represented in our data, in part so that we can specifically identify cancer type-specific features for further investigation (such as in Figure 3C and D). This effect is also exacerbated by our target predictions covering all the compound concentrations. We've added this sentence to the limitations section of the conclusion to reflect this:

> Also, since this work has targeted building a single comprehensive model, it is likely that subtle cancer type specific relationships are not captured such as the relationship between RXRG expression and melanoma (Dempster et al. 2020).

As for Dabrafenib and Vemurafenib, the full model actually has quite a bit of trouble with these two compounds. The R^2 for Dabrafenib is 0.46 (11th percentile), while the R^2 for Vemurafenib is 0.29 (4th percentile).

3. Many of the figures in the paper (e.g. Figure 4, Figure 5) show results/performance metrics summarized across all cell lines and compounds in the dataset. This makes sense for a summary figure, but it seems to me that this could potentially be obscuring variation between cell lines or between compounds. For instance, despite the fact that gene expression does not perform well for viability prediction in general (shown in Figure 4), are there any compounds or any cell lines/tissues of origin where gene expression does outperform kinase inhibition data? If so, is there any biological interpretation for the variation? If this is not the case (i.e. gene expression performs uniformly poorly across cell lines and tissues of origin, and/or kinase inhibition profiles perform uniformly well) this would also be interesting to note.

You make a good point about the summary results in Figure 3 potentially hiding variation in the results across cell lines and compounds, so we've added a panel to sup Figure 2 (now part A). Interestingly there is substantially more variation in model quality across compound vs across cell lines, so it appears that some compounds are just tougher to model correctly. Thank you for the suggestion.
There are no cases where the gene expression data alone outperforms kinase inhibition data alone. We think of the gene expression (and other multiomics data) as providing a background upon which the kinase inhibitors act. We've added this sentence to reflect this finding:

> These model performance differences were also reflected in direct comparisons between individual cell line and compounds, where none of the expression only models outperformed the inhibition state only models.

4. Overall the paper is very clear and well-written. However, as a reader, I would appreciate a bit more clarification earlier on in the paper about exactly what a "kinome inhibition profile" is in the introduction/summary figure since this is central to understanding the paper, especially given that this is a general computational biology journal with readers from diverse fields. Reading through the Klaeger et al. paper, this quote seems like a good summary of why kinome inhibition profiles are useful:

"Owing to the fact many compounds target the structurally and functionally conserved adenosine 5′-triphosphate (ATP)–binding site, polypharmacology (that is, drugs that act on more than one target) is commonly observed. Target promiscuity may have advantageous or detrimental therapeutic consequences."

The idea seems to be that kinase inhibitors can be either targeted or more general, but almost all of them have some "off-target" or polypharmacology effects. Knowing the empirical profile of kinase inhibition for a drug, then, can give you more information than just knowing what it's designed to target. It would be good to put some form of this summary in one of the introductory paragraphs, and/or to explicitly mention exactly what the model features represent (my understanding is that the input is a drug/perturbation x kinase matrix, where each entry quantifies binding/drug interaction strength for the given drug and kinase) since many readers might not be immediately familiar with the data format, or with the observation that kinase inhibitors do not necessarily bind specifically to their intended targets.

This is a very good suggestion and we struggled with how much detail to go into concerning the Klaeger et al data. Your interpretation is correct in that the kinase inhibition state is summarized by a matrix with values for every compound, concentration and kinase that pairs to the degree (0 for full inhibition and 1 for not inhibited) to which each kinase is effected. We've added this section to the introduction to help explain this:

> This work was conducted using a lysate mixture derived from four cell lines which provided a broad representation of the kinome. The results from Klaeger et al. show that many kinase inhibitors have broad target promiscuity and that the kinases targeted by each inhibitor also varies on the basis of the specific compound concentration. Throughout the rest of this paper, we will use the phrase kinase inhibition state to indicate the specific set of kinases targeted by a given compound and to what degree each kinase is inhibited at each concentration.

We've also added this sentence in the results section where we discuss the univariate correlations with kinase inhibition state to further situate the reader:

> The kinase inhibition states are represented as a value lying between zero and one, where zero indicates a fully inhibited kinase and values of one or above indicate that a kinase isn't inhibited.

Hopefully these additions are sufficient to help clarify what we mean by kinase inhibition state.

Minor comments:

5. The authors should be more specific about what form of linear regression is being used in the Methods section. Does the tidymodels default use any form of regularization (e.g. a ridge/LASSO/elastic net penalty), or is it an ordinary least squares fit? It may also be good to describe the default hyperparameter selection strategy for the random forest and XGBoost models - does tidymodels test a variety of parameters using some kind of internal cross-validation, or does it choose a single set of default parameters? Is there any particular reason the authors chose to tune the number of trees in the random forest model and not any other hyperparameters? Hyperparameter selection details can have a considerable effect on performance, at least in my experience, and it is useful to be clear about what was explored/settled on for the final models and why.

The default linear regression engine in tidymodels doesn't use any form of regularization, so we've clarified that in the Model Techniques section. Tidymodels doesn't by default do any sort of hyperparameter tuning, so we opted to first test all the default settings and then attempt to tune the best model. This was partially out of computational necessity, as our ~500,000 data points only allowed us to do limited testing of the initial

models. Reviewer 1 asked if we could try out a Neural Net based method, so we tested TabNet (default settings), but saw similar performance to XGBoost (Figure 3 updated with those results).
As for tuning random forest, we thought we would see the biggest improvements from increasing the number of trees so we tried that first. Even getting these different tree count runs with ~500,000 data points was challenging from a RAM and processor time perspective though. Regardless, we didn't see any dramatic improvements with larger tree counts and so focused on testing out the inclusion of other data types. Reviewer 1 also asked us to try tuning the number of predictors and minimal node size though, so we tried out a range of those values and the results are now in Sup Fig 2C. None of these combinations did much to R^2 or RMSE though, so it looks like the default random forest parameters in tidymodels was robust and nearly optimal for our data.
There are potentially a number of different modeling approaches that could generate an acceptable level of prediction accuracy, including highly-customized models. Here, we chose to limit the range of models tested and instead focus on one of the major novelty points of this work, which was the identification of protein-level inhibition state information as a highly informative piece of data that could provide significant value in modeling efforts leveraging genomic data.
All of these are very good questions and we've added more information about tuning and model type selection in a more extensive Modeling Techniques and Types section in the methods.

6. Methods section, page 18 under "STRING": Ensemble -> Ensembl

This has been fixed in the new version of the paper.

Thank you again for the time you spent reviewing our manuscript.

Sincerely,

Matthew Berginski
Chinmaya Joisa
Brian Golitz
Shawn Gomez
